# NEMAT: An Automated Nonequilibrium Free-Energy Framework for Predicting Ligand Affinity in Membrane Proteins

**DOI:** 10.1021/acs.jcim.5c03089

**Published:** 2026-05-14

**Authors:** Albert Ortega-Bartolomé, Ramon Crehuet

**Affiliations:** † Institute for Advanced Chemistry of Catalonia (IQAC) - CSIC, Barcelona 08034, Catalonia, Spain; ‡ Doctoral Programme in Theoretical Chemistry and Computational Modelling, Universitat de Barcelona, Barcelona 08034, Catalonia, Spain

## Abstract

Quantifying the strength of small-molecule binding to proteins is essential for understanding biological functions and for advancing drug discovery. Computational free-energy methods can predict binding affinities, but membrane proteins remain challenging because of thermodynamic effects of the lipid bilayer. Alchemical free-energy calculations provide state-of-the-art accuracy, and recent nonequilibrium approaches offer improved efficiency and parallelization. However, no existing framework automates these nonequilibrium methods for membrane proteins or enables decomposition of binding free energy into membrane-partitioning and protein-specific components. Nonequilibrium membrane alchemical transformations (NEMAT) is an open-source framework that performs automated nonequilibrium alchemical transformations in water, membranes, and membrane-embedded protein environments. We show that NEMAT reproduces experimental binding energy trends for P2Y_1_ ligands with accuracy comparable to established equilibrium methods. NEMAT provides systematic control over critical simulation parameters to optimize the free-energy estimates. Its ability to dissect membrane and protein contributions supports mechanistically interpretable affinity predictions for membrane-embedded targets.

## Graphical abstract

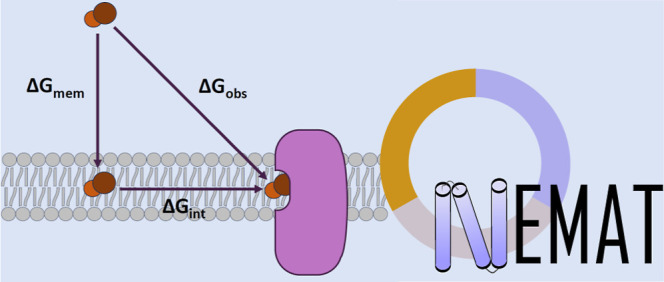

## 1 Introduction

Accurately determining the binding free energy (BFE) of protein–ligand complexes remains a central challenge in computational chemistry, with major implications for drug design and chemical biology in general.^1^ Despite decades of progress, predicting BFEs is still difficult because of the complex balance between enthalpic and entropic effects and the need for extensive sampling.^2^ Even long molecular dynamics simulations often fail to produce reliable or reproducible binding free energies.^3^

Alchemical transformation (AT) methods offer one of the most accurate strategies for computing relative binding free energies (RBFE),^4^ i.e., the difference in BFE of two ligands or two protein variants. These methods estimate RBFE by gradually “mutating” one molecular system into another through a nonphysical—or “alchemical”—pathway. Using a coupling parameter λ ∈ [0, 1], which represents the original and modified molecular systems at λ = 0 and λ = 1, respectively, AT interpolates between molecular Hamiltonians to calculate RBFE, solvation energies, or mutational effects with high precision.^5,6^ AT methods are particularly useful for comparing ligand affinities or studying point mutations in a protein–ligand complex, reducing computational cost while providing mechanistic insight for lead optimization in drug design campaigns.^7–9^

Several rigorous statistical mechanics techniques implement alchemical transformations, including free energy perturbation (FEP)^10^, thermodynamic integration (TI),^11,12^ and nonequilibrium approaches based on Crooks’ fluctuation theorem^13^ and Jarzynski’s equality.^14^ Consequently, several workflows now automate these approaches.^4,15–18^ TI and equilibrium FEP remain widely used for their strong theoretical foundation and consistent performance, but nonequilibrium FEP (NEQ-FEP) has recently become a robust alternative.^19,20^ NEQ-FEP offers similar accuracy with better parallelization and efficiency, especially when combined with estimators such as Bennett’s Acceptance Ratio (BAR).^21^ In 2019, Gapsys et al.^7,22^ released a new NEQ-FEP implementation using GROMACS^23^ and pmx,^24^ making the approach more accessible and reliable.

Despite these advances, alchemical methods remain underused for membrane proteins, particularly with NEQ-FEP.^20^ As membrane proteins represent many drug targets and are central to key biological processes and diseases,^25^ the lack of methods to calculate their RBFE with different ligands hinders progress in medicinal and biological chemistry. Dickson et al. studied GPCRs with membrane-exposed binding pockets,^26^ while Zhang and Im examined GPCRs with internal pockets.^27^ Both studies, performed with TI and AMBER, showed that membranes strongly influence binding energetics when pockets contact the lipid environment. Explicitly including membranes is therefore essential, since ligands may appear to bind tightly merely because they partition into the bilayer rather than engaging in specific protein–ligand interactions.^28–30^ Partitioning can also increase local ligand concentration near the receptor.^31^ Additionally, membrane components such as cholesterol can stabilize proteins or modulate binding.^32,33^ Distinguishing membrane partitioning from specific binding is thus critical for understanding mechanisms and improving ligand selectivity. Including ligand partitioning in free energy calculations allows decomposition of the observed binding energy (Δ*G*_obs_) into membrane insertion (Δ*G*_mem_) and specific interaction (Δ*G*_int_) terms, as illustrated in Figure S2.

To our knowledge, only one study has explicitly decomposed Δ*G*_obs_ using TI,^26^ and no established software or protocol supports this approach for NEQ-FEP. To address this gap, we developed nonequilibrium membrane alchemical transformations (NEMAT), an open-source software that automates setup, execution, and analysis of RBFE calculations for small molecules in three systems: water, membrane, and membrane-embedded protein (MEP) (Figure 1). We also provide a user tutorial and highlight the importance of accounting for membrane affinities when assessing ligand selectivity for membrane-embedded binding sites.

**Figure 1:**
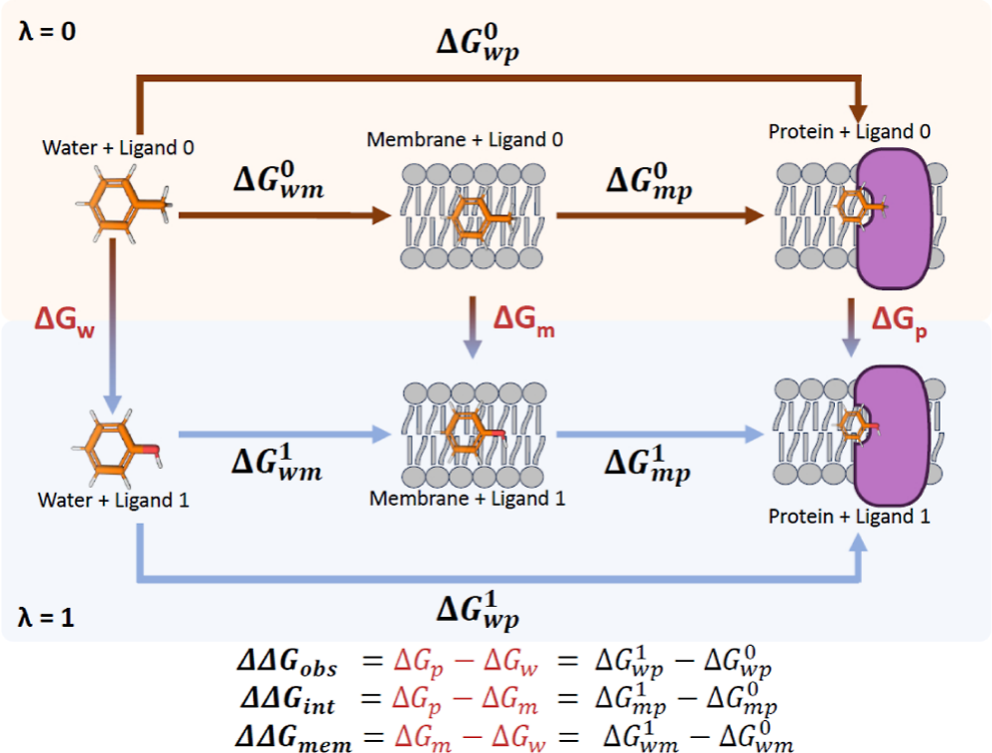
Cycles involved in a NEMAT run. Alchemical transformations consist of transforming ligand 0 to ligand 1 in water, membrane, and protein systems. In the image, ATs are colored in red. Below are the corresponding equations for every part of the cycle. Only the Vertical arrows are simulated. As explained in the text, AT are calculated by 3 replicas, which allows an error estimation that is propagated to ΔΔ*G*_obs_, ΔΔ*G*_int_, and ΔΔ*G*_mem_.

To evaluate NEMAT, we computed RBFE for BPTU and a series of 30 analogues at the P2Y_1_ receptor, a class A GPCR, reproducing the study by Dickson et al.^26^ BPTU binds within a lipid-facing allosteric pocket,^34^ making this system an ideal benchmark where accurate treatment of both membrane partitioning and protein interactions is essential. This benchmark both demonstrates NEMAT in practice and enables a systematic evaluation of the simulation parameters that most affect accuracy in membrane-protein RBFE calculations.

## 2 Methods

### 2.1 The NEMAT Pipeline

NEMAT is a Python3/Bash program that automates nonequilibrium free-energy perturbation (NEQ-FEP) calculations in water, lipid bilayers, and membrane-embedded protein (MEP) environments. It implements the preparation of equilibrium production trajectories and nonequilibrium transitions as well as their numerical analysis. Its workflow, based on the tutorial code for FEP using pmx^35^ (Figure 2), combines pmx for the preparation of the dual topologies used in FEP with GPU-accelerated GROMACS simulations. Ligand parameters and topologies are generated through ACPYPE using the GAFF2 force field, which has been tested for NEQ methods.^36^ Final free energies are obtained from the work distribution, as implemented in BAR. It balances flexibility and fine-grained control with an ease of use.

**Figure 2:**
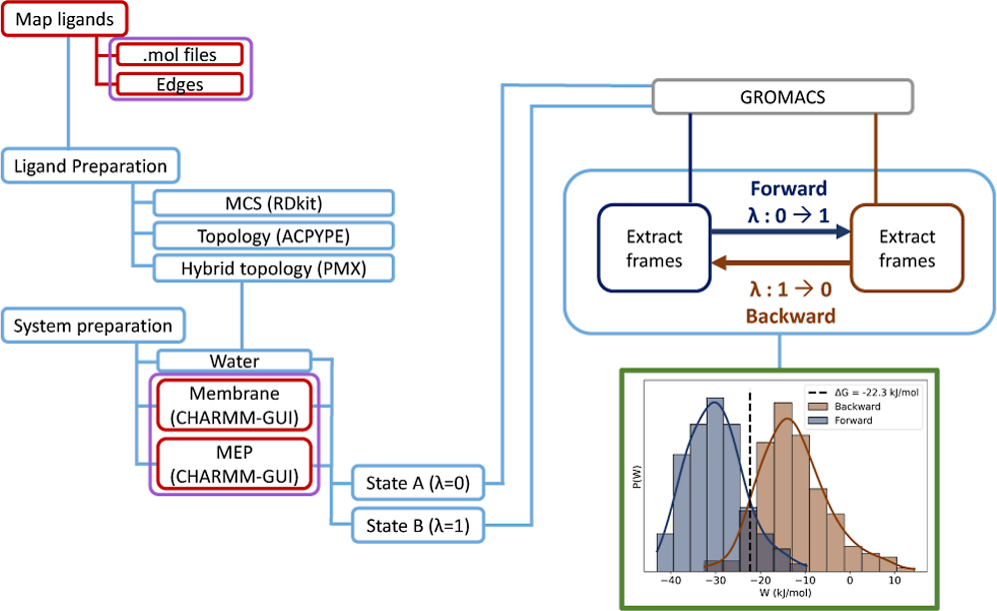
Simple schematics of the NEMAT usual workflow. Red labels highlight the actions the user performs, purple labels indicate the inputs provided to NEMAT, blue elements show the operations executed by NEMAT, and green elements represent the program’s outputs.

NEMAT automates:

- Generation of hybrid topologies via maximum common substructure (MCS) mapping.
- Setup of alchemical transformations across the chosen edges.
- Execution of NEQ-FEP simulations in parallel replicas.
- Analysis of relative binding free energies (ΔΔ*G*).

pmx prepares ligands for alchemical transformations and has demonstrated state-of-the-art accuracy in published benchmarking studies using open-source tools.^7,37^ It performs especially well for small- to medium-sized protein–ligand systems.

### 2.2 System Preparation

Following Figure 2, users first prepare the MEP, the membrane, and the ligands. NEMAT begins from systems built without the ligands; CHARMM-GUI is recommended for preparing MEP and membrane systems,^38–41^ though any well-defined GROMACS coordinates and topology files are accepted.

Ligands are provided as mol2 files. NEMAT automatically selects alchemical atoms through an MCS search between ligand pairs using pmx. Shared atoms remain across the alchemical pathway, while unique atoms become dummy atoms that disappear or appear along the λ-interpolation. This ensures consistent hybrid topologies with both real and dummy atoms for smooth free-energy transitions.^24,42^ pmx relies on ACPYPE^43^ to supply force–field parameters. ACPYPE wraps Antechamber to generate GROMACS-compatible topologies for small molecules under GAFF2.

### 2.3 Running NEMAT

After system preparation, NEMAT performs free-energy simulations for the three environments—water, membrane, and membrane-embedded protein (MEP)—using the GROMACS software. Following the thermodynamic cycles in Figure 1, relative free energies are derived: ΔΔ*G*_obs_ (from MEP and water), ΔΔ*G*_int_ (from membrane and MEP), and ΔΔ*G*_mem_ (from membrane and water). These magnitudes are obtained from the calculated Δ*G*_*p*_, Δ*G*_*m*_, and Δ*G*_*w*_ obtained from the work-distributions with BAR, which also estimates their errors (see the equations in Figure 1).

To ensure robust statistics, more than one replica (the default is 3) is recommended for each system. Each simulation begins with energy minimization, followed by NPT-restrained equilibration. For MEP and membrane systems, equilibration proceeds in six stages with progressively weaker restraints, ending with an unrestrained equilibration to stabilize lipids as suggested by the CHARMM-GUI input generator.^44^

After equilibration, an NPT production trajectory is run from which a series of alchemical transformations (from now on, transitions, since the transformation is a transition from a state A (ligand 1) to a state B (ligand 2)) are used to compute the RBFE, as done in NEQ-FEP^45^ (Figure 3). The recommended length of this trajectory is discussed in the Supporting Information. The reported RBFE (RBFEs) are averaged across replicas.

**Figure 3:**
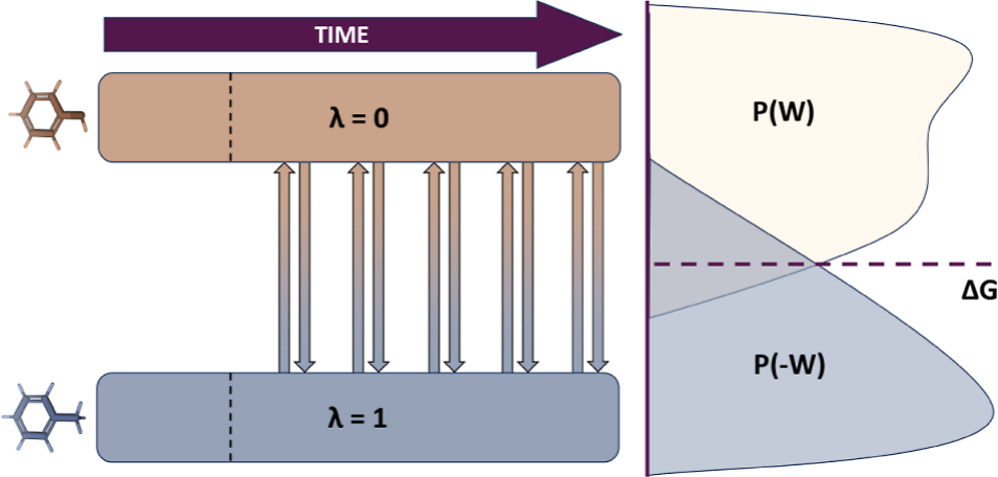
Free energy calculation with nonequilibrium FEP. The molecular dynamics trajectories for the two ligands (λ = 0 and λ = 1) are split into an equilibration phase (before the dashed line) and a production phase. From the production phases, fast nonequilibrium transitions are performed forward (from 0 to 1) and backward (from 1 to 0). The free energy is obtained from the work distribution of the transitions using different estimators.

Because NEMAT uses NEQ-FEP, short transitions capture the instantaneous work changes during ligand swapping. Several estimators can produce the free energy difference from the work distribution obtained. The simplest and seminal estimator is the Jarzynski equality (eq 1:^14^)­

1 $$e^{-\Delta G/kT}=e^{-\left\langle W/kT\right\rangle }$$

However, the Bennett Acceptance Ratio (BAR) method^21^ provides a more reliable estimator by optimally weighting forward and reverse work distributions.^46^ NEMAT calculates free energies using Jarzynski’s equality, its Gaussian approximation, and BAR, but reports only BAR-derived results due to their superior convergence when bidirectional sampling is available.

Average work values derive from overlapping forward and reverse work distributions;^3^ adequate overlap is essential for numerical stability, and forward and backward distributions that are very distant are a sign of concern. But our analysis reveals that minimal overlap is enough to produce small errors in ΔΔ*G* with the BAR estimator (see Figure S14). Following best practices,^47^ users should calibrate parameters for each target rather than rely on fixed settings. We recommend validating a small subset of edges first, tuning transition durations and parameters until they meet overlap/uncertainty criteria.

When multiple replicas are run, NEMAT computes a Gaussian-weighted average of the free energy values of every replica. Using a Gaussian-weighted average assigns a weight to each replica value based on its proximity to the others. If the free energy of a replica is much different than the others, then its weight is significantly lower, and so is its contribution to the final energy value. The *i*-th replica weight (ω_*i*_) is given by

$$\omega_{i}=\underset{j}{\sum }e^{(\Delta G_{i}-\Delta G_{j})/2\sigma^{2}}$$ 2

where Δ*G*_*i*_ and Δ*G*_*j*_ are the computed free energies for the *i*-th and *j*-th replicas and σ modulates the contribution of the dispersion. It is automatically set to the rule-of-thumb value^48^ such that σ = std­(Δ*G*_*j*_)*n* ^–1/5^, where std­(Δ*G*_*j*_) is the standard deviation of the free energy values and *n* is the number of replicas. The user can set σ → ∞ (i.e., 10^9^), so that the average will be arithmetic (equal weights for every replica). Then, the final values are computed with the following average

3 $$\Delta G_{env}=\frac{\sum_{i}\omega_{i}\Delta G_{i}}{\sum_{i}\omega_{i}}$$

where *i* is the replica number and env ∈ [w, m, p] (see (Figure 1)).

### 2.4 A Case Example: P2Y_1_ with BPTU Analogs

We used 14 antagonist ligands reported by Dickson et al.,^26^ as Δ*G*_mem_ was calculated only for this subset. Chao et al.^49^ determined *K*_*i*_ using radioligand binding assays, from which Δ*G* was derived as Δ*G* = – *RT*ln­(*K*_*i*_) at 298.15 K. This Δ*G* corresponds to Δ*G*_obs_. As in ref 26, we divided the 14 ligand set into two groups (11a-like BPTU analogues and 6a-like BPTU analogues, Figures S8, S9), and aligned them with the BPTU reference using the NEMAT align.sh script based on RDkit. A detailed description of the different systems setup can be found in the Supporting Information.

## 3 Results and Discussion

### 3.1 Performance of NEMAT on the P2Y_1_–BPTU Ligand Series

We evaluated NEMAT on the set of BPTU analogues previously characterized for antagonistic activity at the P2Y_1_ receptor. Correct prediction of the sign and magnitude of RBFE (ΔΔ*G*_obs_) is critical for guiding lead optimization, as negative values indicate improved affinity and positive values indicate decreased binding.

We used the experimental ΔΔ*G*_obs_ values reported by Chao et al.^49^ to benchmark NEMAT. In addition, we compared the results with an Amber-based equilibrium free-energy perturbation study performed by Dickson et al.^26^ In this paper, the authors present two values for ΔΔ*G*_obs_: one computed using TI from a single edge (which is equivalent to ΔΔ*G*_mem_ + ΔΔ*G*_int_, Figure 1) and one computed with multiple edges using the Arsenic software (now Cinnabar^50^). For the purpose of the present work, we will compare directly energies arising from the same edge, thus ΔΔ*G*_obs_^*D*^ = ΔΔ*G*_mem_+ΔΔ*G*_int_ in Table 1.

**Table 1:** Comparison of Results^a^

edges	ΔΔ*G*_obs_^exp^(kcal/mol)	ΔΔ*G*_obs_^*D*^(kcal/mol)	ΔΔ*G*_obs_ (kcal/mol)	ΔΔ*G*_int_ (kcal/mol)	ΔΔ*G*_mem_ (kcal/mol)
11a → 11b	–2.8 ± 0.5	–6.0 ± 0.5	–5.6 ± 0.2	–3.0 ± 0.5	–2.6 ± 0.4
11a → 11f	–1.5 ± 0.5	–1.4 ± 0.8	–2.27 ± 0.16	–0.5 ± 0.3	–1.8 ± 0.3
11a → 11c	–2.4 ± 0.6	–2.4 ± 0.9	–3.1 ± 0.4	–0.8 ± 0.5	–2.3 ± 0.3
11a → 1	–2.0 ± 0.4	–1.8 ± 0.8	–1.85 ± 0.16	0.6 ± 0.2	–2.46 ± 0.16
6a → 6i	–1.0 ± 0.4	–2.7 ± 0.2	–2.6 ± 0.2	1.0 ± 0.3	–3.6 ± 0.2
6a → 6f	–0.1 ± 0.5	–1.5 ± 0.1	0.76 ± 0.08	–0.4 ± 0.3	1.2 ± 0.3
6a → 6h	–0.8 ± 0.7	–0.8 ± 0.3	–0.67 ± 0.12	1.06 ± 0.19	–1.73 ± 0.17
6a → 6m	0.6 ± 0.5	–0.7 ± 0.9	2.7 ± 0.2	2.7 ± 0.3	0.0 ± 0.2
6a → 6g	–0.5 ± 0.6	–1.6 ± 0.3	–3.4 ± 0.4	–3.1 ± 0.6	–0.3 ± 0.5
6a → 6L	–1.2 ± 0.7	–0.0 ± 0.4	1.18 ± 0.19	0.9 ± 0.2	0.3 ± 0.2
6a → 6j	0.4 ± 0.7	–3.1 ± 0.3	–0.1 ± 0.3	1.0 ± 0.3	–1.0 ± 0.2
6a → 6n	0.7 ± 0.5	–0.3 ± 0.5	2.46 ± 0.15	0.34 ± 0.19	2.13 ± 0.14

a Table presenting the results obtained experimentally by Chao et al.^49^ (ΔΔ*G*_obs_^exp^), the results obtained by Dickson et al. without using Arsenic (the ones that come from adding ΔΔ*G*_int_ and ΔΔ*G*_mem_; ΔΔ*G*_obs_^*D*^),^26^ and by NEMAT for the 11a group.

For the 11a-centered star map (Figure S8), NEMAT correctly reproduces the experimentally observed ranking of ligands relative to 11a (Table 1, top). All predicted ΔΔ*G*_obs_ values show the correct direction of affinity change, indicating that the workflow reliably distinguishes affinity gains from losses. We observe a similar trend for the 6a-centered series (Figure S9), with correct directional predictions in 87.5% of ligand pairs (taking into account that the experimental values for 6a → 6f and 6a → 6j are essentially 0; Table 1, bottom). These results demonstrate that NEMAT provides accurate prioritization guidance for structure–activity relationship (SAR) exploration.

Beyond predicting bound-state affinities, NEMAT also estimates ΔΔ*G*_int_, the free-energy difference between the membrane and the receptor-bound states. This metric separates improvements in receptor specificity from changes arising primarily from altered membrane partitioning. NEMAT results have a lower correlation with Dickson et al. for ΔΔ*G*_int_ and ΔΔ*G*_mem_ than for ΔΔ*G*_obs_ but lacking experimental data for these components it is not possible to assess which method gives more accurate results. To the extent that the lipid bilayer is a hydrophobic environment, one would expect a correlation of ΔΔ*G*_mem_ and logP values of the ligands. To this aim, we estimated logP with RDKit and plotted them with respect to ΔΔ*G*_mem_ in Figure S11. Although the absolute values differ, the lipophilicity-derived free energy changes correctly reproduce the sign of ΔΔ*G*_mem_, indicating qualitative agreement. This correlation is similar to the one obtained by Dickson et al.,^26^ and in both cases, one should not expect a strong correlation as the lipid bilayer is more heterogeneous and anisotropic than 1-octanol.

When ligands with high flexibility are inserted into the membrane, equilibration of their different conformations can be slow, and this can affect the calculated ΔΔ*G*_mem_ and, consequently, also ΔΔ*G*_int_. As we do not have experimental values to assess the importance of this effect, we recommend that users inspect the production trajectory in the membrane to assess whether the ligand samples multiple conformational states and are properly positioned. If the ligand fails to do so, extending the simulation time of the production phase in the membrane may partially mitigate this limitation. Nevertheless, in some cases, the use of production trajectories generated with enhanced sampling methods may be necessary, an aspect that will be addressed in future work.

To obtain absolute binding free energies (Δ*G*, Figure 4), we used a maximum likelihood estimator provided by the *cinnabar* package along with the experimental values provided by Chao et al.^49^ Pairwise ΔΔ*G* correlations can be found in Figure S10a (for the 11a subgroup) and Figure S10b (for the 6a subgroup) along with the star map used to calculate the transformation (Figures S8, S9). NEMAT reproduced the experimental trends with an RMSD of 1.69 kcal· mol^–1^ (MUE of 1.39 kcal· mol^–1^ with a correlation coefficient of *r* ^2^ = 0.44 and Kendall τ = 0.42; p-value = 0.037) These metrics are similar to the ones reported by Dickson et al.^26^ Indeed, we obtain a stronger correlation between our results and those of Dickson et al. with an *r* ^2^ value of 0.57 and Kendall τ = 0.57 (*p*-value = 0.021 Figure S13). Therefore, NEMAT achieves predictive accuracy comparable to state-of-the-art alchemical workflows while incorporating NEQ-FEP, membrane systems, fewer user-defined steps, and maintaining full reproducibility through its automated pipeline.

**Figure 4:**
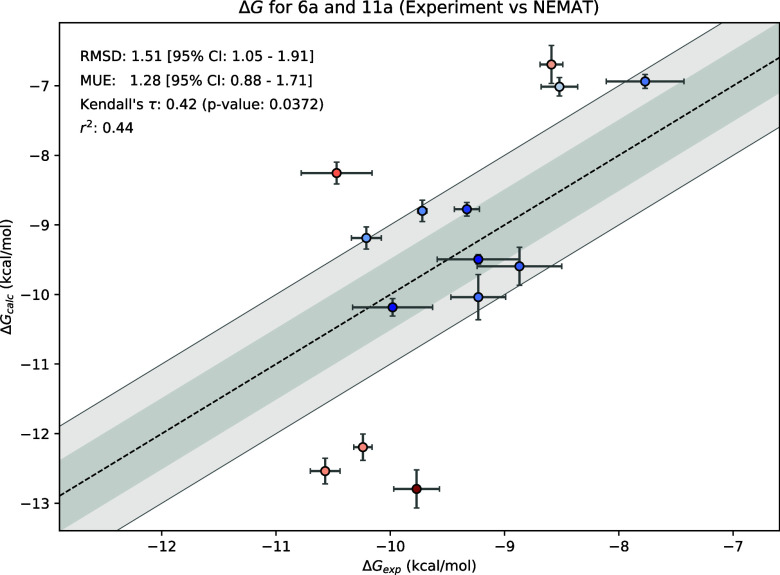
Predicted absolute binding free energies (Δ*G*) for the 14 studied BPTU analogues using cinnabar. The figure also presents the values of the RMSD and MUE for the samples, along with the 95% confidence interval.

On the basis of Supporting Information Section 2, the benchmark was conducted using a production dynamic of 20 ns and 50 transitions evenly spaced, lasting 100 ps. These values are the defaults when using NEMAT but can be easily modified. However, it is reasonable to start testing your system with these values. With these values, most of the uncertainty comes from the independent replicas; therefore, increasing the number of replicas is the most practical way to increase the statistical accuracy.

## 4 Conclusions

In this work, we introduced NEMAT, the first automated pipeline for performing nonequilibrium alchemical free-energy calculations in explicit water, membrane, and membrane-embedded protein environments. By combining pmx-based hybrid topology generation with GPU-accelerated GROMACS simulations, NEMAT provides a fully reproducible workflow that decomposes observed affinities (ΔΔ*G*_obs_) into membrane partitioning (ΔΔ*G*_mem_) and receptor-interaction (ΔΔ*G*_int_) components. Applied to the P2Y_1_ receptor and a series of BPTU analogues, NEMAT reproduced experimental trends with accuracy comparable to established equilibrium approaches while maintaining excellent computational efficiency and parallel scalability.

Although NEMAT offers a practical and broadly applicable framework, several challenges remain. The optimal NEQ-FEP settings can vary by system, and parametrization with current force fields may limit performance for flexible or chemically complex ligands. In addition, heterogeneous membrane compositions and slow lipid or protein motions can introduce sampling difficulties that the present implementation does not fully address.

These limitations highlight opportunities for future development, including broader force field support, enhanced sampling strategies, and improved handling of complex or asymmetric membranes. Such extensions would increase the robustness and expand the range of systems that can be modeled reliably.

In summary, NEMAT fills a critical gap in the free-energy landscape by enabling fast, reproducible, and membrane-aware nonequilibrium calculations for ligand binding. Its ability to separate membrane and protein contributions provides new mechanistic insight into ligand selectivity at lipid-facing pockets, and continued development will help establish NEQ-FEP as a routine tool in membrane-protein drug discovery.

## Supplementary Material



## Data Availability

Data and Software Availability the NEMAT software is available as open-source under a CC BY-NC-ND 4.0 license and can be accessed at the NEMAT GitHub repository (https://github.com/QTC-IQAC/NEMAT). The repository includes the complete source code, installation instructions, and a tutorial that reproduces the calculations reported in this study and illustrates the application of the workflow to new membrane protein systems. All input files, analysis scripts, and processed data for the P2Y_1_ benchmark are available in the repository, with raw MD trajectories available from the authors upon request.

## Notes

Derivation of the Δ*G*_obs_ thermodynamic decomposition; benchmark results for production run lengths (5, 20, and 50ns) and transition durations (50, 100, and 200 ps) across water membrane, and membrane-embedded protein environments; analysis of the effect of transition count (10–100) on free energy estimates and work-distribution overlap; star maps for the11a- and 6a-centered BPTU analogue series; pairwise ΔΔ*G*_obs_ correlation plots versus experimental values for both ligand subgroups; lipophilicity (logP) versus predicted ΔΔ*G*_mem_; and comparison of NEMAT versus Dickson et al.’s absolute and relative binding free energies using cinnabar (PDF)
