# Supplementary material for: NEMAT: An Automated Nonequilibrium Free-Energy Framework for Predicting Ligand Affinity in Membrane Proteins
Source: J Chem Inf Model. 2026 May 14;66(10):5573–9. doi: 10.1021/acs.jcim.5c03089 (PMC13213831; doi:10.1021/acs.jcim.5c03089)
Supplement: Supplementary file 1 [file ci5c03089_si_001.pdf]

Supporting Information for Publication.

NEMAT: An Automated Non-Equilibrium Free-Energy Framework for Predicting Ligand Affinity in Membrane Proteins

Albert Ortega-Bartolomé[1,2], **Ramon Crehuet**[1,*]

[1]Institute for Advanced Chemistry of Catalonia (IQAC) - CSIC
[2]Doctoral programme in Theoretical Chemistry and Computational Modelling, Universitat de Barcelona, Barcelona, Spain

* **Email**: ramon.crehuet@iqac.csic.es

Contents

1 Detailed description of the P2Y$_1$ with BPTU analogs system setup S2

2 Choosing NEMAT parameters S4

3 Definition of the Different Binding Free Energies S5

4 Discussion on the derivation of $\Delta G_{\mathbf{obs}}$ S6

5 Testing numbers S7

6 Study on the number of transitions vs error size and overlap effects S9

7 Star maps S11

8 Comparison with experimental data of the RBFE values obtained. S12

9 Study of lipophilicity vs predicted $\Delta\Delta G_{mem}$ S13

10 Comparison of the values obtained by NEMAT versus Dickson et al. S14

11 Study of the BAR error with the overlap score S16

Supplementary Note 1: Detailed description of the P2Y$_1$ with BPTU analogs system setup

A. Membrane-embedded protein (MEP) system: the P2Y$_1$- POPC membrane complex. The P2Y$_1$– BPTU complex (4xnv, 2.2 Å resolution (11)) was downloaded from the OPM database (6). The P2Y$_1$ protein was protonated at pH 7.4 using the CHARMM-GUI input generator. The OPM structure lacks the ICL3 domain due to disorder; however, this region does not affect ligand binding, so the missing residues were not modeled. Termini were capped with ACE and NME. Amber ff19SB was used for the protein (9). A POPC (1-Palmitoyl-2-oleoyl-sn-glycero-3-phosphocholine) bilayer (9.5 x 9.5 nm, 233 lipids) was constructed after aligning the protein along the Z-axis. Lipid parameters used Lipid21 (4). The system contained 0.15 M NaCl (56 Na^+, 76 Cl^-) and 20,939 TIP3P water molecules (5). Waters inside the helical bundle were added with Dowser++ (7) to improve realism. Fig. S1 shows the final complex.

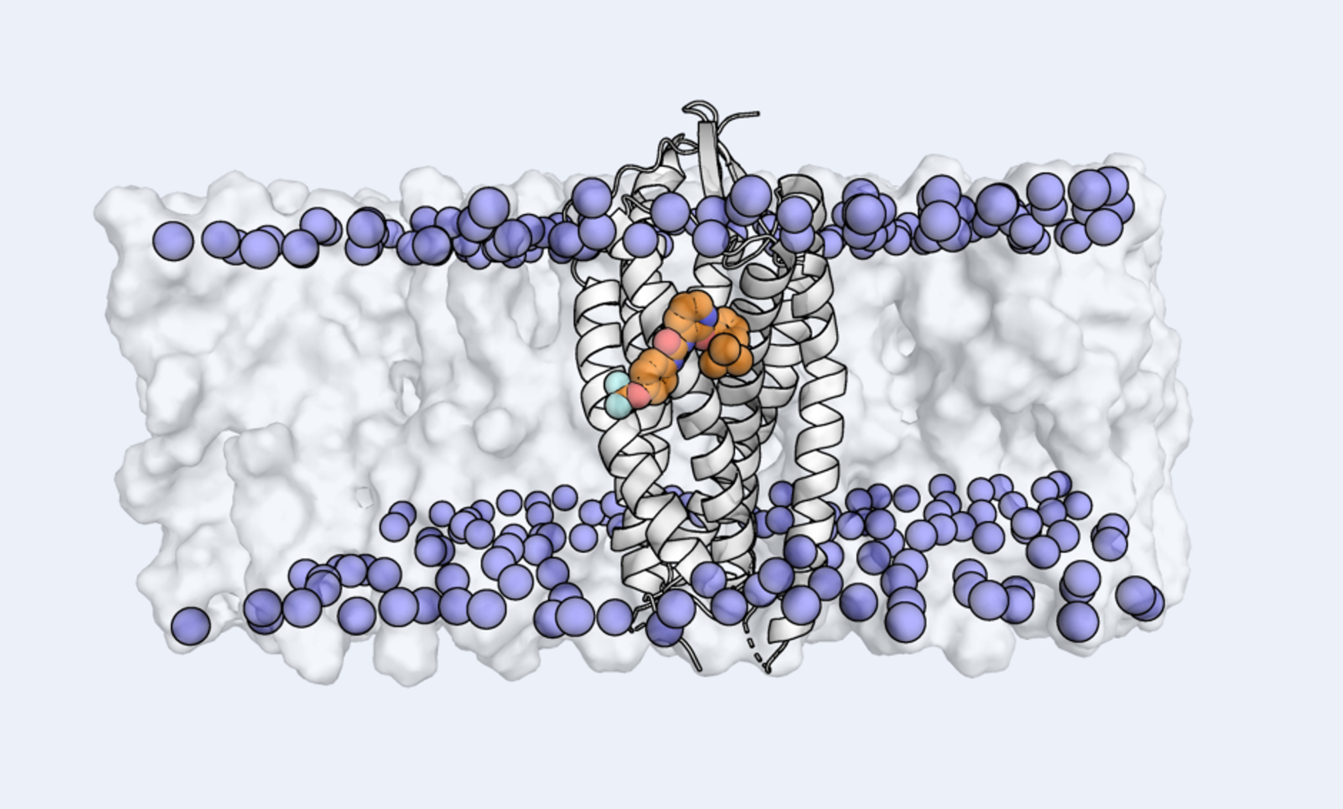

Fig. S1. Membrane-Embedded P2Y$_1$ Receptor. Representation of the equilibrated membrane-embedded P2Y$_1$ receptor including the BPTU ligand (orange) in the binding pocket. Water molecules and ions are not expressed for clarity. The polar heads of the lipids in the bilayer are represented in blue.

B. Membrane system. A POPC bilayer (4 × 4 nm, 48 lipids) was built with 0.15 M NaCl (5 ions each type) and 2,132 TIP3P water molecules.

C. Small molecules: BPTU analogues. The P2Y$_1$–BPTU complex was aligned with the P2Y$_1$-POPC membrane complex, and the ligand was extracted as a reference for FEP alignment. As Dickson *et al.* already defined the mapping of the candidate ligands (star maps at Fig. S8, S9), that selection step was omitted. Each ligand was solvated in a 4.5 nm cubic box with ≈1,990 water molecules and ionized with NaCl (six ions per type for neutral ligands) using AM1-BCC charges. Then, pmx performs alchemical mapping.

D. Running NEMAT. All free-energy simulations were performed with GROMACS 2024.2 using the NEMAT NEQ-FEP protocol.

Three replicas were run to improve convergence. All systems underwent a 5,000-step energy minimization (tolerance 1,000) via steepest descent, followed by a NPT equilibration of 1.5 ns for the systems containing membrane and 0.5 ns for the ligands in water, using the C-rescale barostat. For membrane and MEP systems, equilibration included six stages with progressively weaker heavy atom restraints (1,000 → 100 kJ/mol/Å^2 for bilayer; 4,000 → 50 kJ/mol/Å^2 for MEP) and a final unrestrained phase. We set the restraint force values following CHARMM-GUI's recommendations. For all the simulations, we used an inverse friction constant of $\tau_t = 1.0$. For a single system, we checked that values between 0.5 and 2 resulted in similar work

distributions that resulted in free energy estimates with much less dispersion within the same replica with different frictions than the dispersion arising from different replicas.

After equilibration, 20 ns NPT production runs were performed, saving 200 frames at regular intervals. Starting from 5 ns, 50 evenly spaced alchemical transitions were executed with 100 ps extensions, using a 2 fs timestep and stochastic Langevin dynamics. These transitions were implemented via GROMACS free-energy options, and all simulations used identical protocols and parameters. The specific parameters used are provided in the mdp files in the GitHub repository.

Supplementary Note 2: Choosing NEMAT parameters

A. Computational Efficiency and Parallelization. Because alchemical transformations in each environment (water, membrane, and MEP) are independent across systems and replicas, the NEMAT workflow is highly parallelizable. When all replicas were executed simultaneously, the total wall time per edge was determined primarily by the MEP system, which was the largest. This parallel structure permitted rapid exploration of ligand pairs without serial computational bottlenecks.

Furthermore, the *multidir* option can be used to further enhance the performance during the transitions calculation (decreasing approximately by 15 times the wall clock time using an A100 GPU with respect to simulating the transitions in serial).

Completion of all transitions and analyses required approximately 15 GB of storage per edge when using three replicas per environment.

B. Length of the production. To evaluate the influence of production length on the accuracy of the non-equilibrium transitions, we tested three simulation durations: 5, 20, and 50 ns per replica (Fig. S3, S4, S5). At a temperature of 298 K, the analysis was performed for 100 evenly spaced transitions, each lasting 200 ps. Both protocols were applied to a representative subset of ligand pairs in the three environments (water, membrane, and MEP). The resulting free energy estimates showed more replica dispersion for the 50 ns productions, making them less reliable. As for the 5 and 20 ns productions, there is no considerable difference between the mean value of one production and the other. However, using a 20 ns production spaces more the transitions, making sure there is no correlation if they are contiguous. Consequently, all subsequent production simulations were performed using a 20 ns trajectory, which provided equivalent accuracy at substantially reduced computational cost while maintaining an acceptable error.

C. Number of transitions. To assess the influence of the number of non-equilibrium transitions on the calculated free energies, we performed tests using 10, 20, 25, 33, 50, 80, and 100 evenly spaced transitions per edge of 200 ps, while maintaining a temperature of 298 K and a production run of 20 ns (the first transition starting from 5 ns). The results, summarized in Tab. S1 and Fig. S7, show that increasing the number of transitions beyond 50 had little impact on the predicted relative free energies (maximum deviations $\leq$0.05 kcal$\cdot$mol^{-1} across all tested edges) and the predicted errors were also small. This is because even a small number of transitions produces very similar overlaps between the resulting work distributions, as can be seen in Fig. S7. Based on this analysis, subsequent production simulations were performed using 50 uniformly spaced transitions, minimizing the number of transitions while maintaining a reasonable error.

D. Length of the transitions. To evaluate the effect of transition length on the accuracy and convergence of the non-equilibrium work distributions, we tested 50 ps, 100 ps, and 200 ps transition durations for representative ligand pairs in all three environments (water, membrane, and MEP) and using three replicas. While maintaining a temperature of 298 K and a production run of 20 ns, as shown in Supplementary Fig. S3, S4, S5, transitions of 50 ps produced inconsistent RBFE estimates and showed forward and reverse distributions centered at very distant values, indicating inadequate sampling and poor convergence. In contrast, 100 ps and 200 ps transitions yielded highly consistent RBFEs, differing by less than 0.2 kcal$\cdot$mol^{-1} and exhibiting reduced statistical uncertainty. Therefore, a transition length of 100 ps was chosen as the default in NEMAT, providing an optimal balance between accuracy and computational efficiency.

Supplementary Note 3: Definition of the Different Binding Free Energies

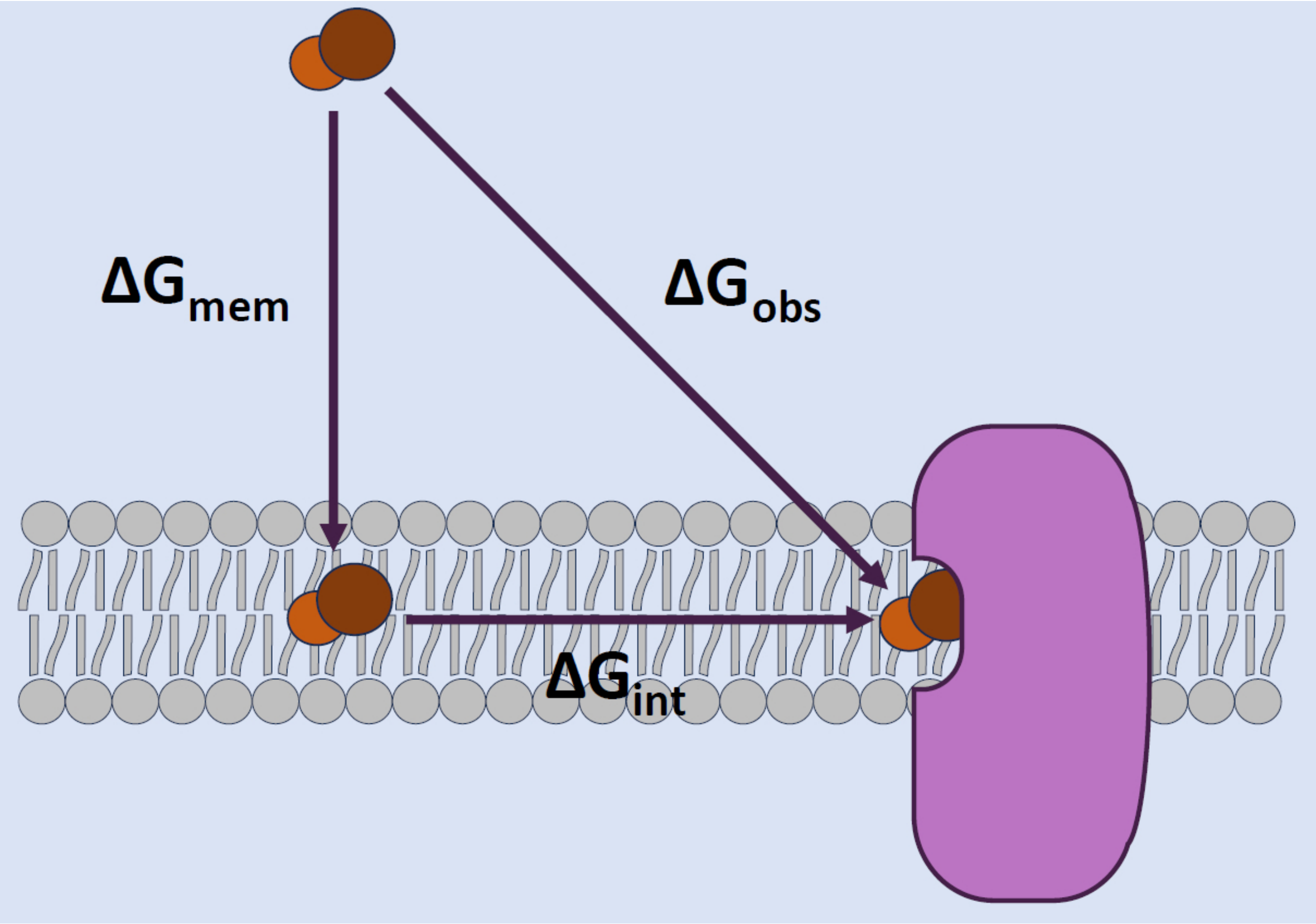

Fig. S2. Definition of the Different Binding Free Energies. The binding free energy of a ligand to a membrane protein (ΔG_{obs}) can be decomposed into two steps: the ligand binding to the membrane (ΔG_{mem}) plus the binding from the membrane to the protein (ΔG_{int}).

Supplementary Note 4: Discussion on the derivation of ΔG_{obs}

In membrane-embedded protein systems, ligand binding is further complicated by the physicochemical role of the lipid bilayer, which introduces additional kinetic and thermodynamic factors. For example, before a ligand can bind to a membrane protein's site, especially an extra-helical or lipid-exposed site, it must first partition into the membrane (2). Furthermore, membrane partitioning increases the local concentration of the ligand near the receptor, enhancing the observed binding affinity, even if the ligand's true interaction with the binding site is weak (1).

Another factor that complicates the affinity of a membrane-embedded receptor is that the ligand's affinity to the membrane may be similar to its affinity to the receptor, leading to a misperception of the true affinities with the protein. As stated in (3, 8, 10), if there is binding when a membrane is involved, there is a clear correlation between drug concentration and the observed binding affinity. This means that the aggregated association rate constant of the ligand (K_{aa}) is concentration dependent:

$$K_{aa} = [L] \cdot K_a + K_d \tag{4}$$

Where K_a is the association rate constant and K_d is the dissociation rate constant, which reflects the true stability of the binding. Then, knowing that association is proportional to the concentration of the ligand, but k_d is not, Sykes *et al.* (8) observed a direct correlation between k_a and a new constant which reflects the membrane binding K_{mem}. Then we can obtain an estimation of the "real" binding of the ligand (K_{int}) with:

$$pK_{\text{int}} = pK_{\text{obs}} - pK_{\text{mem}} \tag{5}$$

If alchemical transformations are performed in water, in the membrane, and the protein + membrane system, a thermodynamic cycle can be created such that the relations among relative free energies, based on EQ. 5, become:

$$\Delta G_{\text{obs}} = \Delta G_{\text{mem}} + \Delta G_{\text{int}} \tag{6}$$

Such that ΔG_{obs}, ΔG_{mem} and ΔG_{int} are the relative free energy between the protein + membrane system and water, between the membrane and the protein + membrane system and between the membrane and water system respectively as can be observed in Fig. S2.

Supplementary Note 5: Testing numbers

About the length of the production and the transitions.

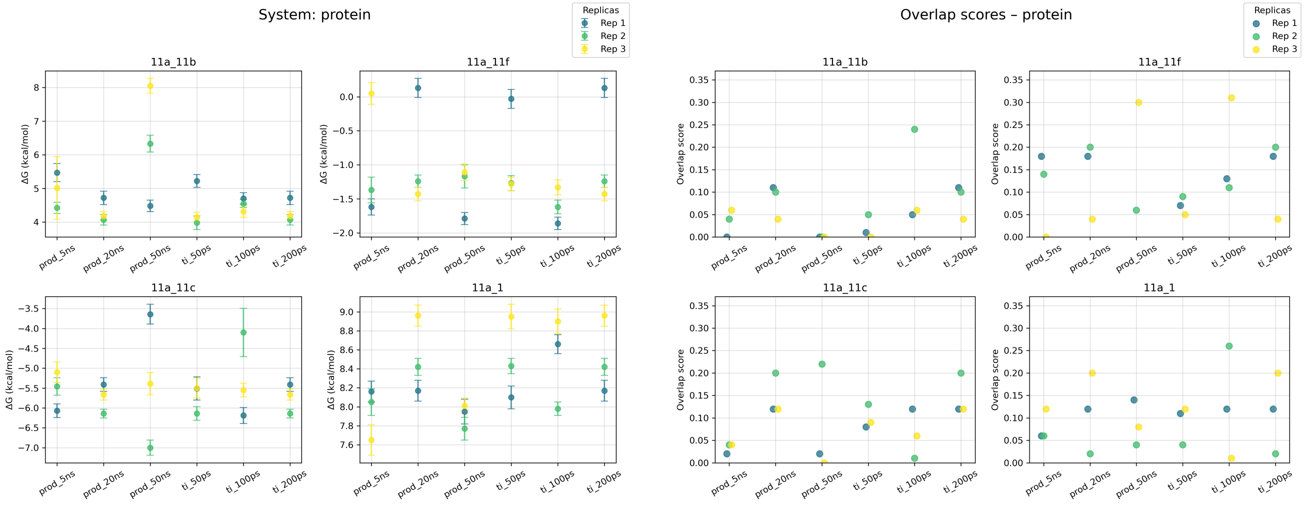

(a) Computed ΔG_p (Fig. 1) for the 11a ligand subsystem. **(b)** Overlap between forward and backwards trajectories of the 11a ligand subsystem.

Fig. S3. Tests for the MEP system of overlap and results. The results of 3 replicas are plotted. We studied how the results vary with changes in the lengths of the production runs (prod) and the transitions (ti).

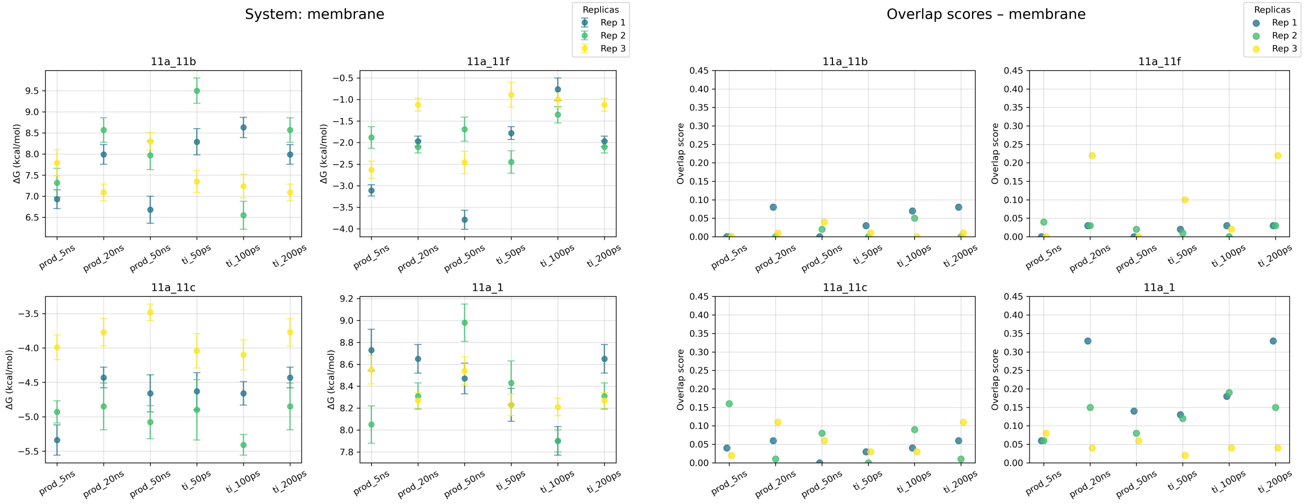

(a) Computed ΔG_m (Fig. 1) for the 11a ligand subsystem. **(b)** Overlap between forward and backwards trajectories of the 11a ligand subsystem.

Fig. S4. Tests for the membrane system of overlap and results. The results of 3 replicas are plotted. We studied how the results vary with changes in the lengths of the production runs (prod) and the transitions (ti).

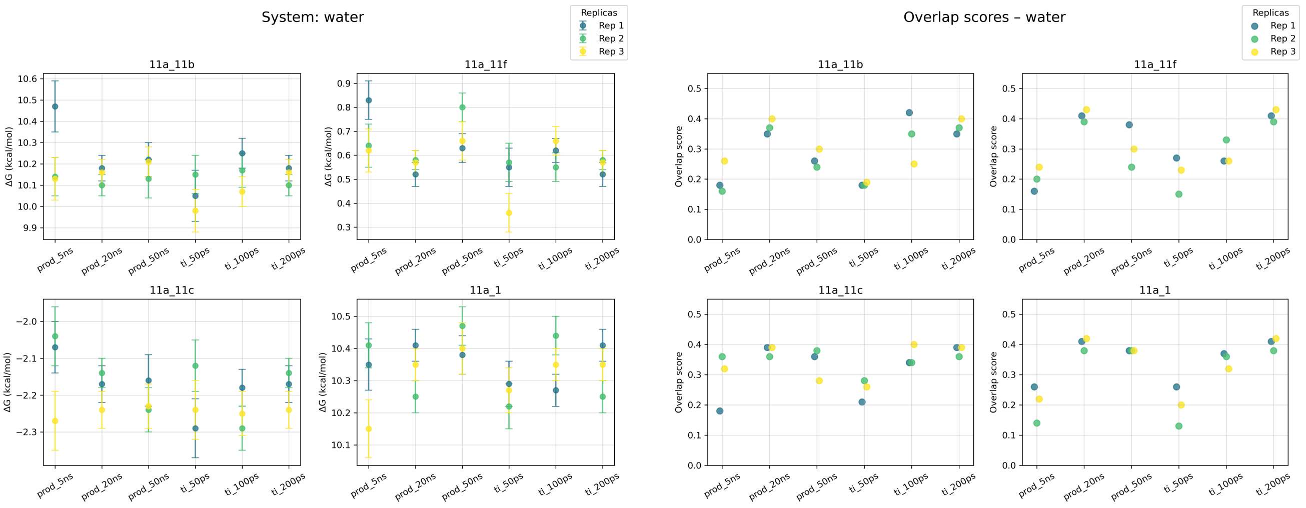

(a) Computed ΔG_w (Fig. 1) for the 11a ligand subsystem. **(b)** Overlap between forward and backwards trajectories of the 11a ligand subsystem.

Fig. S5. Tests for the water system of overlap and results. The results of 3 replicas are plotted. We studied how the results vary with changes in the lengths of the production runs (prod) and the transitions (ti).

Edge	100	80	50	33	25	20	10
11a → 11b	-5.98 ± 0.13	-6.0 ± 0.2	-5.9 ± 0.3	-5.9 ± 0.3	-5.9 ± 0.4	-5.4 ± 0.4	-5.3 ± 0.5
11a → 11f	-2.00 ± 0.11	-2.1 ± 0.3	-1.9 ± 0.4	-2.0 ± 0.4	-2.0 ± 0.4	-2.1 ± 0.6	-1.6 ± 0.7
11a → 11c	-3.42 ± 0.14	-3.4 ± 0.2	-3.6 ± 0.3	-3.5 ± 0.4	-3.5 ± 0.4	-4.1 ± 0.4	-4.3 ± 0.4
11a → 1	-1.39 ± 0.11	-1.34 ± 0.10	-1.31 ± 0.13	-1.35 ± 0.15	-1.23 ± 0.13	-1.4 ± 0.2	-1.2 ± 0.3

Table S1. Results obtained using different numbers of transitions. Values obtained from a single replica for the $\Delta\Delta G_{\text{obs}}$ of the 11a group. The values correspond to a production of 20 ns (starting transitions from 5 ns) and transitions lasting 200 ps. The transitions are evenly spaced.

Supplementary Note 6: Study on the number of transitions vs error size and overlap effects

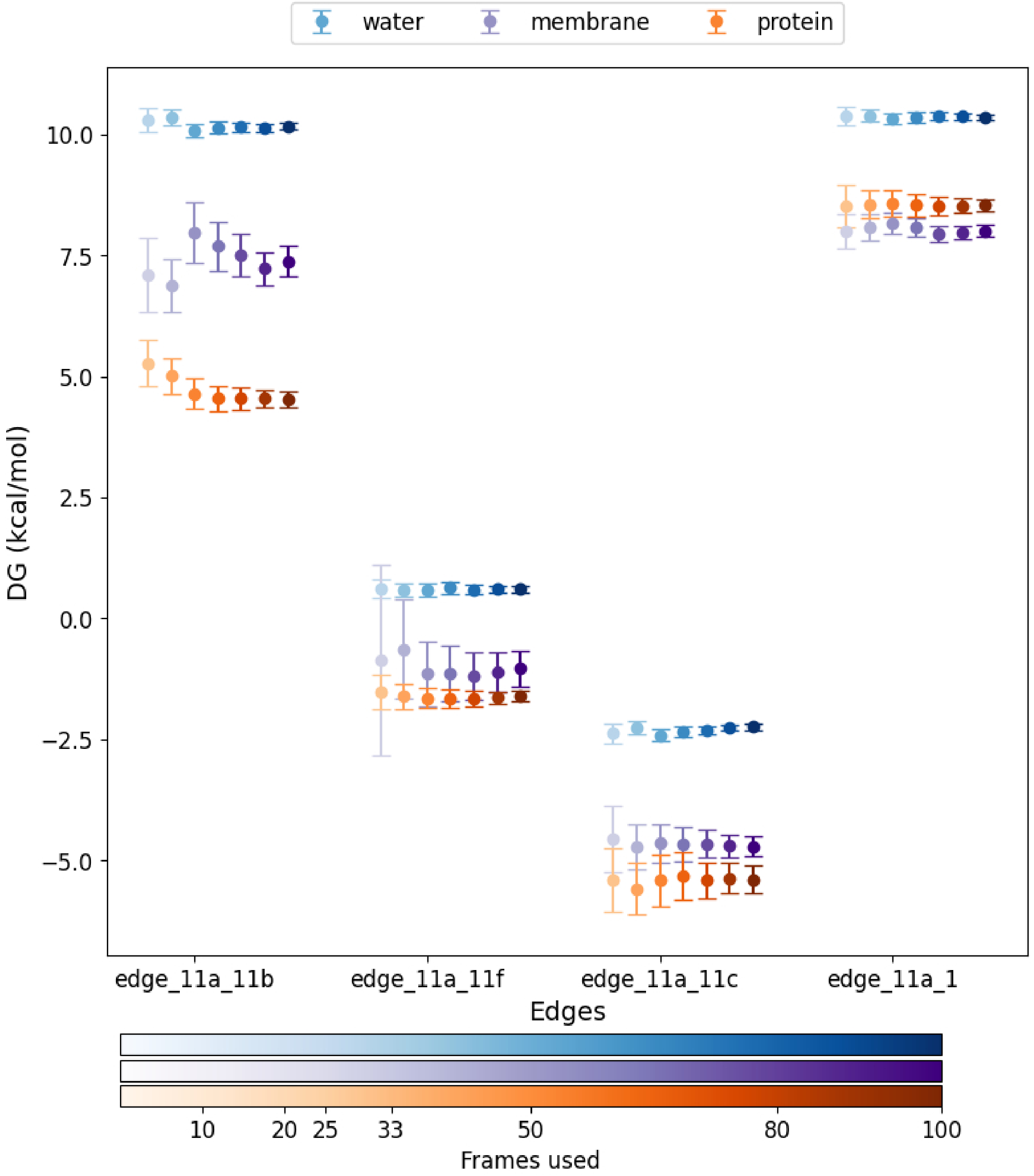

Fig. S6. Example of the effect of using fewer transitions. Reducing the number of transitions drastically reduces the sampling of uneven behavior and increases its associated error, potentially leading to different results.

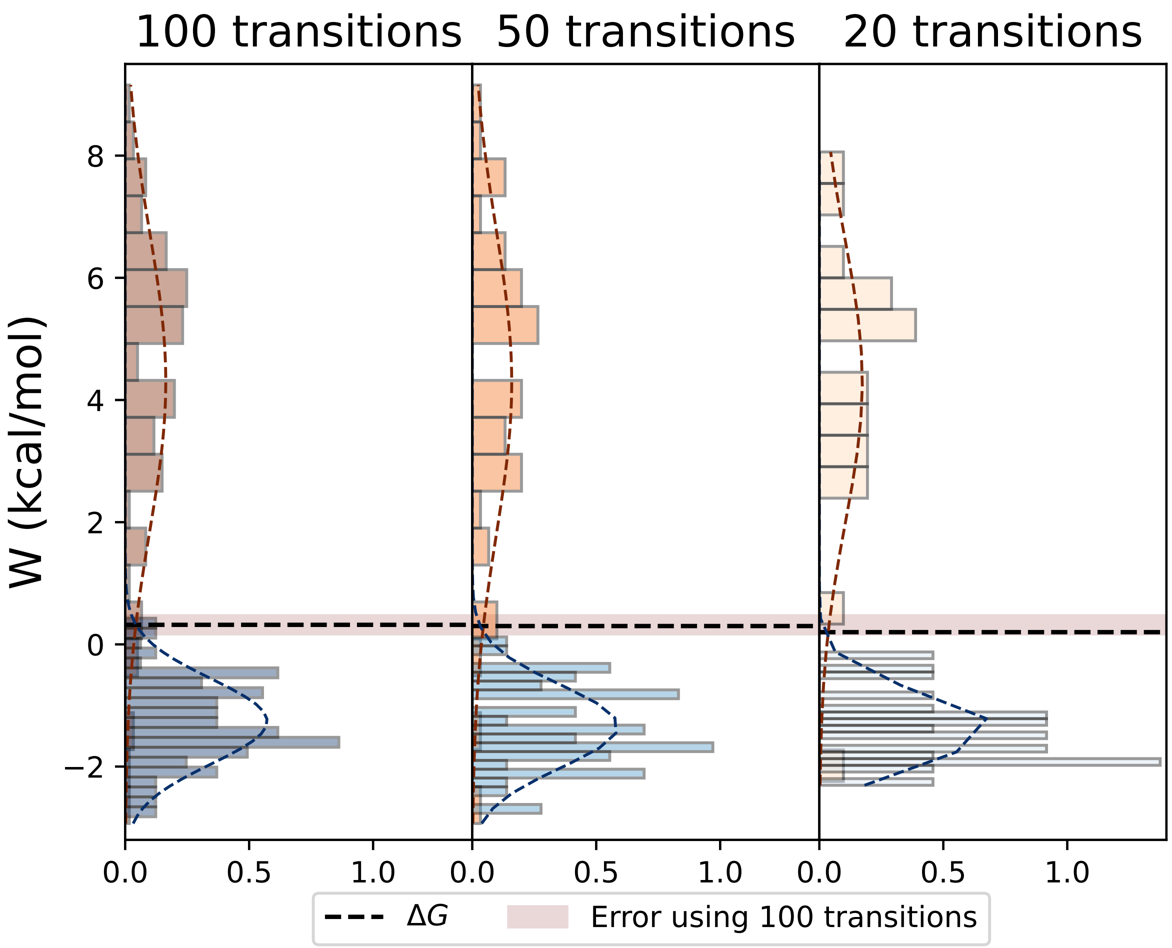

Fig. S7. Study of overlap between forward and backwards trajectories of the 11a ligand with the membrane subsystem. In the example, we can observe the distribution change when reducing the number of transitions. There is a small shift in the intersection point of the distributions. The corresponding value of ΔG using 100, 50, and 20 transitions is displayed with a black-dashed line. The red shaded area indicates the error of ΔG when using 100 transitions (which is the most precise value). As can be seen in the figure, the shift of the energy from using 100 or 50 transitions is negligible. However, further decreasing the number of transitions makes the results more imprecise.

Supplementary Note 7: Star maps

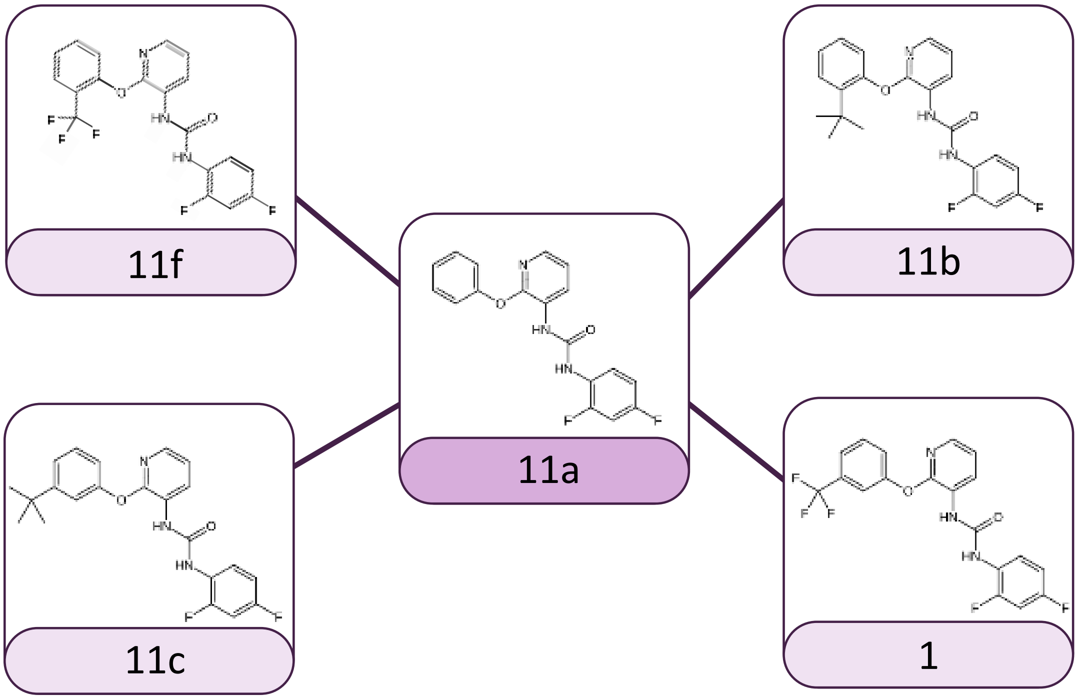

Fig. S8. Start map centered on 11a for the BPTU analogues.

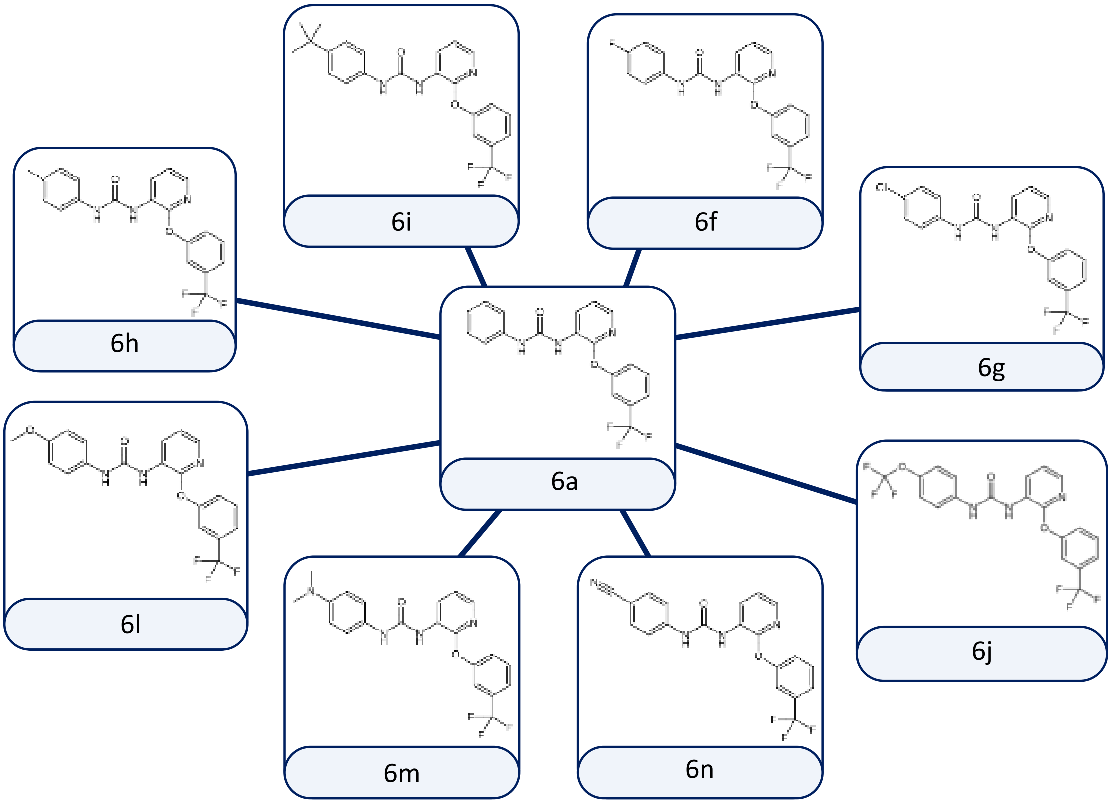

Fig. S9. Start map centered on 6a for the BPTU analogues.

Supplementary Note 8: Comparison with experimental data of the RBFE values obtained.

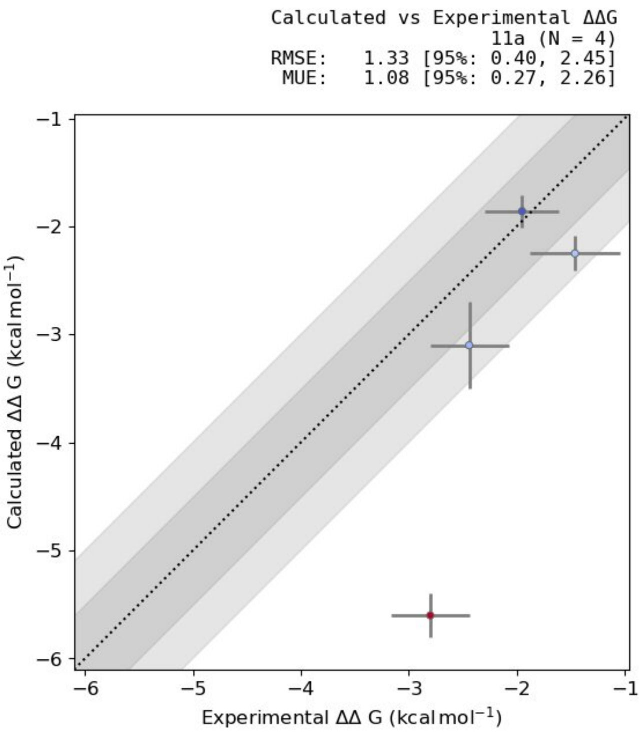

(a) Comparison of the computed $\Delta\Delta G_{\mathrm{obs}}$ (Calculated $\Delta\Delta G$) versus the corresponding experimental value (Experimental $\Delta\Delta G$) for the BPTU analogues centered on 11a (top values of Tab. 1). Plotted with *cinnabar*.

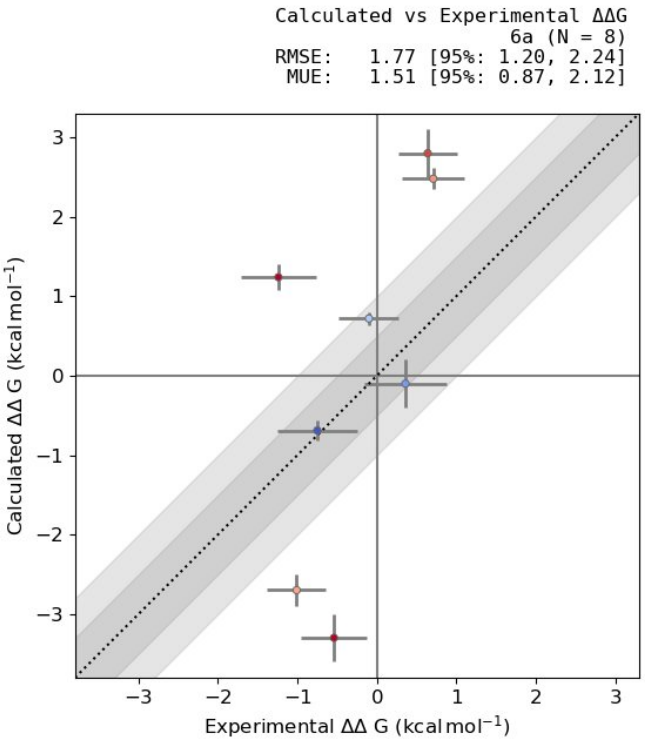

(b) Comparison of the computed $\Delta\Delta G_{\mathrm{obs}}$ (Calculated $\Delta\Delta G$) versus the corresponding experimental value (Experimental $\Delta\Delta G$) for the BPTU analogues centered on 6a (bottom values of Tab. 1). Plotted with *cinnabar*.

Fig. S10. Comparison of the experimental values of $\Delta\Delta G_{\mathrm{obs}}$ with the ones obtained using NEMAT.

Supplementary Note 9: Study of lipophilicity vs predicted $\Delta\Delta G_{mem}$

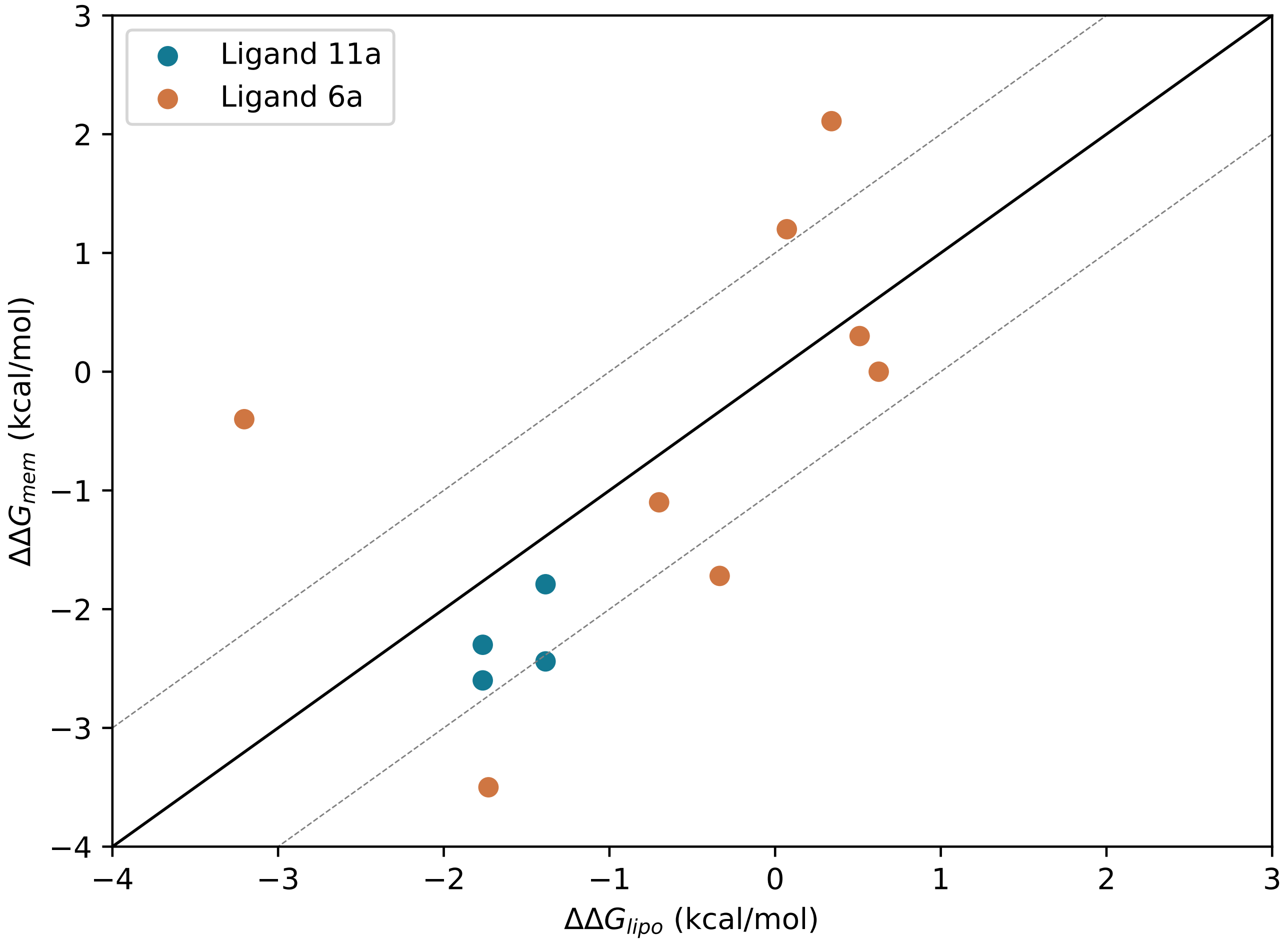

Fig. S11. Lipophilicity versus NEMAT. The lipophilicity was calculated using $\Delta\Delta G_{\text{lipo}} = -2.303 \cdot RT \cdot \Delta \log_{10} P$ where P is partition coefficient.

Supplementary Note 10: Comparison of the values obtained by NEMAT versus Dickson et al.

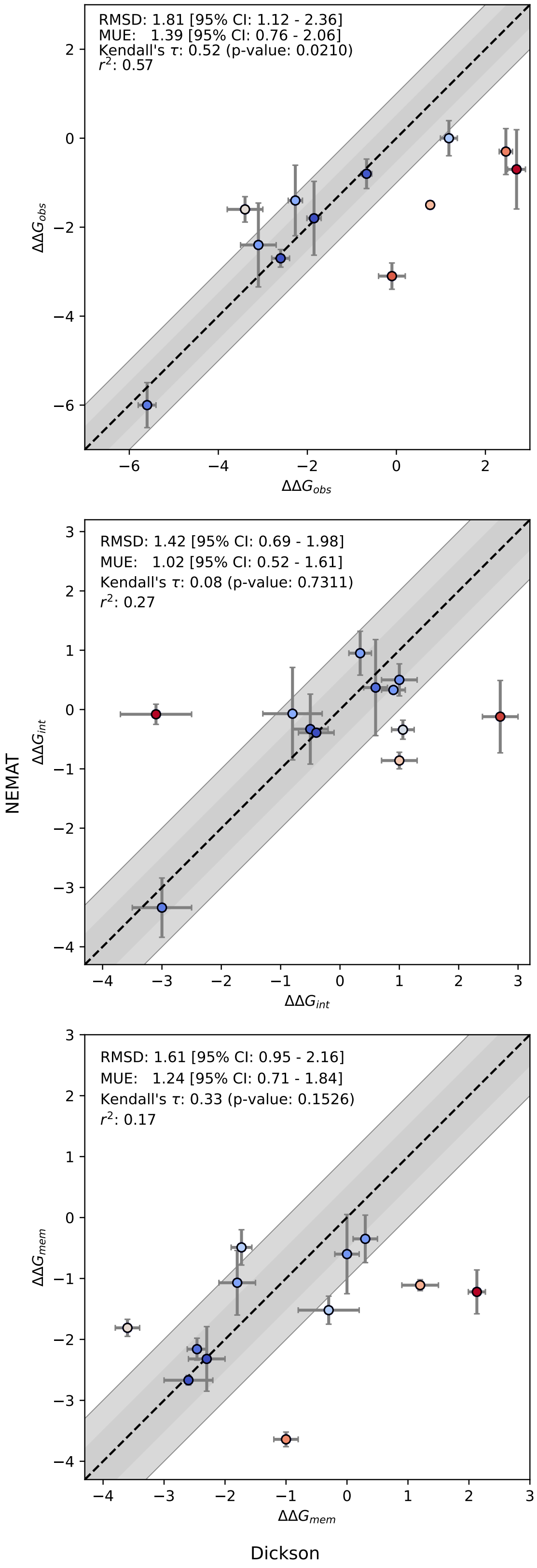

Fig. S12. RBFE values of NEMAT versus Dickson et al. (2). The Y-axis corresponds to the NEMAT results and the X-axis to the results obtained by Dickson et al. using AMBER TI.

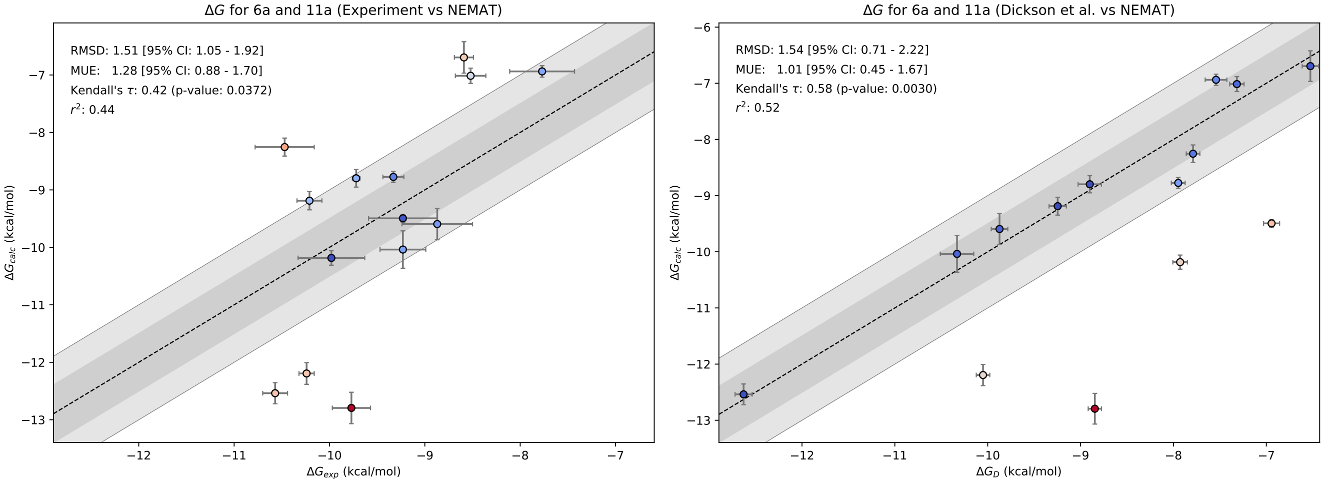

Fig. S13. ABFE values of NEMAT compared with experimental values and Dickson et al. (2) computed values using *cinnabar* (before, Arsenic).

Supplementary Note 11: Study of the BAR error with the overlap score

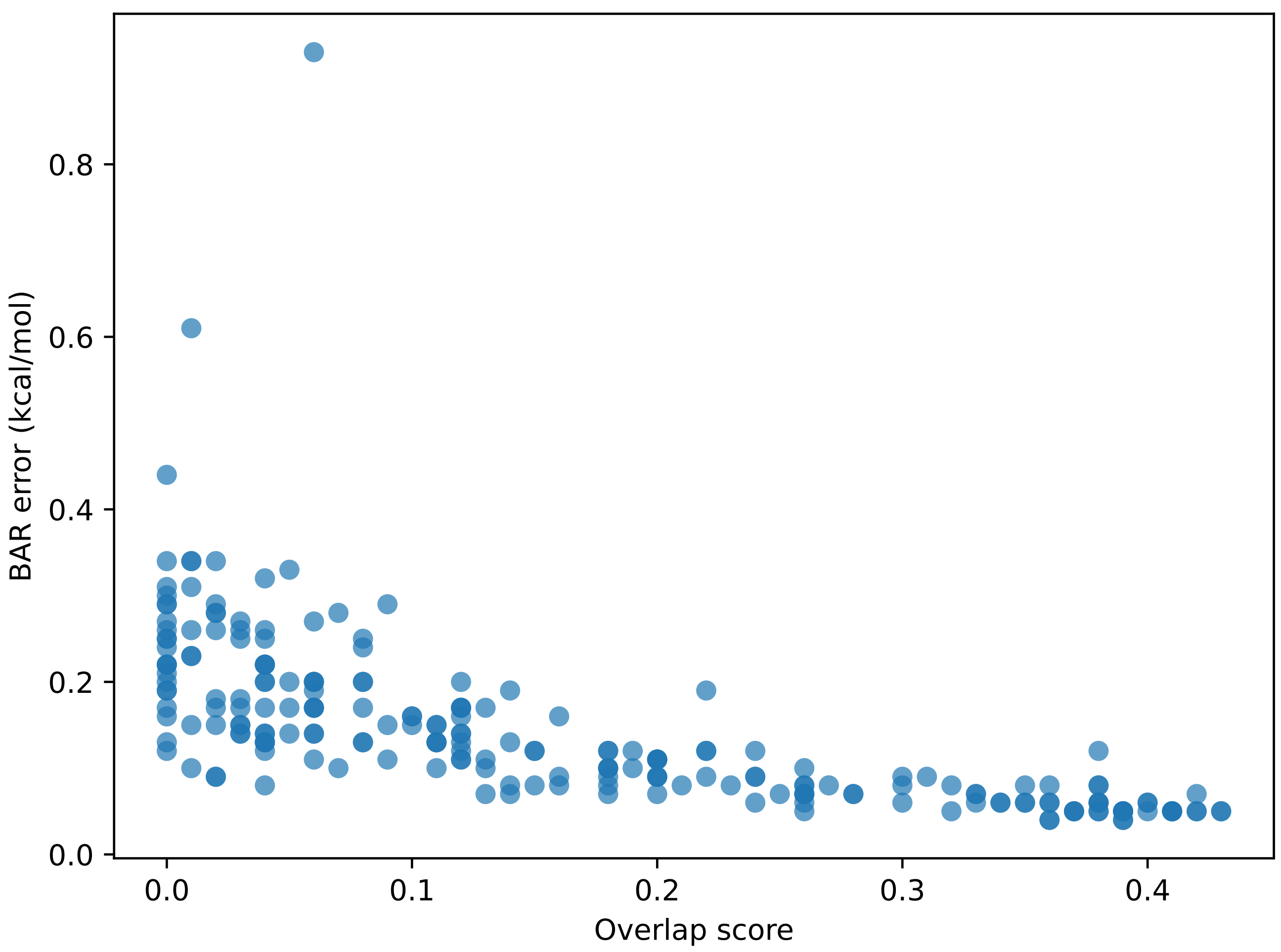

Fig. S14. BAR error with the overlap score. Plotted using all the data of Fig. S3,S4,S5.

Supplementary Information References

1. Allison Pearl Barkdull, Matthew Holcomb, and Stefano Forli. A quantitative analysis of ligand binding at the protein-lipid bilayer interface. *Communications Chemistry*, 8(1):89, March 2025. Publisher: Nature Publishing Group.
2. Callum J. Dickson, Viktor Hornak, and Jose S. Duca. Relative Binding Free-Energy Calculations at Lipid-Exposed Sites: Deciphering Hot Spots. *Journal of Chemical Information and Modeling*, 61(12):5923–5930, December 2021.
3. Callum J. Dickson, Viktor Hornak, Camilo Velez-Vega, Daniel J. J. McKay, John Reilly, David A. Sandham, Duncan Shaw, Robin A. Fairhurst, Steven J. Charlton, David A. Sykes, Robert A. Pearlstein, and Jose S. Duca. Uncoupling the Structure-Activity Relationships of β2 Adrenergic Receptor Ligands from Membrane Binding. *Journal of Medicinal Chemistry*, 59(12):5780–5789, June 2016.
4. Callum J. Dickson, Ross C. Walker, and Ian R. Gould. Lipid21: Complex Lipid Membrane Simulations with AMBER. *Journal of Chemical Theory and Computation*, 18(3):1726–1736, March 2022.
5. William L. Jorgensen, Jayaraman Chandrasekhar, Jeffry D. Madura, Roger W. Impey, and Michael L. Klein. Comparison of simple potential functions for simulating liquid water. *The Journal of Chemical Physics*, 79(2):926–935, July 1983.
6. Mikhail A. Lomize, Andrei L. Lomize, Irina D. Pogozheva, and Henry I. Mosberg. OPM: orientations of proteins in membranes database. *Bioinformatics (Oxford, England)*, 22(5):623–625, March 2006.
7. A. Morozenko and A. A. Stuchebrukhov. Dowser++, a New Method of Hydrating Protein Structures. *Proteins*, 84(10):1347–1357, October 2016.
8. David A. Sykes, Cheryl Parry, John Reilly, Penny Wright, Robin A. Fairhurst, and Steven J. Charlton. Observed drug-receptor association rates are governed by membrane affinity: the importance of establishing "micro-pharmacokinetic/pharmacodynamic relationships" at the β2-adrenoceptor. *Molecular Pharmacology*, 85(4):608–617, April 2014.
9. Chuan Tian, Koushik Kasavajhala, Kellon A. A. Belfon, Lauren Raguette, He Huang, Angela N. Migues, John Bickel, Yuzhang Wang, Jorge Pincay, Qin Wu, and Carlos Simmerling. ff19SB: Amino-Acid-Specific Protein Backbone Parameters Trained against Quantum Mechanics Energy Surfaces in Solution. *Journal of Chemical Theory and Computation*, 16(1):528–552, January 2020.
10. Georges Vauquelin. On the 'micro'-pharmacodynamic and pharmacokinetic mechanisms that contribute to long-lasting drug action. *Expert Opinion on Drug Discovery*, 10(10):1085–1098, October 2015.
11. Dandan Zhang, Zhan-Guo Gao, Kaihua Zhang, Evgeny Kiselev, Steven Crane, Jiang Wang, Silvia Paoletta, Cuiying Yi, Limin Ma, Wenru Zhang, Gye Won Han, Hong Liu, Vadim Cherezov, Vsevolod Katritch, Hualiang Jiang, Raymond C. Stevens, Kenneth A. Jacobson, Qiang Zhao, and Beili Wu. Two disparate ligand binding sites in the human P2Y1 receptor. *Nature*, 520(7547):317–321, April 2015.
